# From Computational Prediction to *In Vivo* Experimental Validation: A Translational AI Pipeline for Anthracycline-Induced Cardiotoxicity

**DOI:** 10.21203/rs.3.rs-10594011/v1

**Published:** 2026-08-13

**Authors:** Manqi Li, Jing Wei, Shuteng Niu, Lu Kang, Ping Zhu, Joerg Herrmann, Xiaolei Xu, Cui Tao

**Affiliations:** a Department of Biostatistics and Data Science, School of Public Health, The University of Texas Health Science Center at Houston, Houston, TX, USA.; b Department of Artificial Intelligence and Informatics, Mayo Clinic, Jacksonville, FL, USA.; c Department of Biochemistry and Molecular Biology, Mayo Clinic, Rochester, MN, USA.; d Department of Cardiovascular Medicine, Mayo Clinic, Rochester, MN, USA.

**Keywords:** anthracycline-induced cardiotoxicity, drug repurposing, knowledge graph, large language model, zebrafish, cardio-oncology

## Abstract

Anthracycline-induced cardiotoxicity (AIC) remains a dose-limiting complication of cancer treatment, often leading to irreversible heart failure, yet effective cardioprotective therapies remain limited. Graph-driven drug repurposing offers a rapid pathway to identify cardioprotective therapies, but different computational models often generate divergent candidate lists that exceed experimental validation capacity. We applied a novel “Union-then-Filter” framework to prioritize therapeutics for AIC using AI-based approaches and evaluated candidates in zebrafish models. Three state-of-the-art graph learning models (TxGNN, CompGCN, and RLR) were trained on the PrimeKG to generate an initial candidate pool. A Large Language Model (LLM) agent automated evidence synthesis across 4,286 PubMed abstracts for the top 50 drugs from each model. Candidates were stratified using dual decision criteria and filtered against existing clinical trials. The pipeline narrowed 150 initial candidates to four novel candidates: Cysteine, Dasatinib, Tranilast, and Tretinoin. Prospective *in vivo* validation was subsequently conducted using an adult AIC (aAIC) zebrafish model. Phenotypic screening via echocardiography revealed that Cysteine, Tranilast, and Tretinoin significantly restored ejection fraction (RF) (overall one-way ANOVA: *F*(4,95) = 47.66, *p* < 0.0001; Tukey's post hoc: *p* < 0.01 for all three treatments vs. DOX+ control) and normalized cardiac remodeling markers in doxorubicin-treated subjects (Kruskal-Wallis: *χ*^2^(4) = 17.21, *p* = 0.0018; Dunn’s post hoc: *p* < 0.01 for all three comparisons vs. DOX control). This study demonstrates the translational utility of an LLM-integrated AI pipeline for drug repurposing and highlights zebrafish AIC as an efficient bridge between *in silico* prediction and *in vivo* phenotypic validation in cardio-oncology.

## Introduction

Anthracyclines, such as doxorubicin (DOX), are the cornerstone of chemotherapy for a wide spectrum of malignancies, including lymphomas, leukemias, and breast cancer^1^. Their clinical utility is compromised by cumulative, dose-dependent cardiotoxicity, which can progress to irreversible cardiomyopathy and heart failure^2^. As the cancer-survivor population grows, the need for cardioprotective interventions increases^3^. Currently, no FDA-approved mechanism-based drug treats AIC patients who already manifest cardiac dysfunction, typically months or years after their chemotherapy^4,5^. Dexrazoxane, the only FDA-approved compound for AIC, is restricted to patients during chemotherapy^6^. Consequently, there is an urgent need to identify safe, effective, and readily available therapeutics to mitigate AIC, especially for patients who already manifest cardiac dysfunction. Accordingly, the present study is directed at the treatment of AIC rather than its prophylactic prevention during anthracycline exposure.

Drug repurposing offers a time- and cost-efficient alternative to *de novo* drug development^7^. While the integration of Artificial Intelligence (AI) has revolutionized this field, significant challenges remain^8,9^. Graph Neural Networks (GNNs) and network-based algorithms often generate vast, divergent lists of candidate drugs based on different topological assumptions^9,10^. This “prediction explosion” creates a bottleneck, as experimental validation remains costly and time-consuming^11,12^. Previously, we addressed this challenge in the context of Alzheimer’s Disease by developing a framework that harmonizes diverse graph model outputs using an LLM for automated evidence synthesis^8^. Here, we adapt this “Union-then-Filter” framework to cardio-oncology, leveraging the predictive power of state-of-the-art (SOTA) graph models (TxGNN^9^, CompGCN^13^, and RLR^10,14^) and the reasoning capabilities of LLMs to generate a prioritized list of candidates for experimental verification.

While AIC models have been well established in mice for mechanistic studies, they have limited throughput for assessing large numbers of candidate drugs. Zebrafish has emerged as an attractive alternative vertebrate model^15^. Both embryonic AIC (eAIC) and adult AIC (aAIC) zebrafish model have been established, which can be used to experimentally assess therapeutic efficacy of a much larger number of compounds^15^. Cardioprotective effects of Dexrazoxane has been faithfully recapitulated in the eAIC model, supporting the conservation of this experimental platform. While the throughput is higher in eAIC zebrafish model, the aAIC zebrafish model more faithfully recapitulates progressive pathogenesis in human AIC, enabling elucidation of time-dependent mechanisms, such as autophagy^16^. Top drugs from the eAIC model can be validated in the aAIC model, and further in a mouse AIC model, as recently demonstrated for FDA-approved autophagy-activating drugs^16^. Therefore, we adopted this zebrafish eAIC-aAIC platform to assess candidate drugs suggested by AI.

To prove the effectiveness of a translational decision framework by integrating zebrafish AIC models with complementary graph-learning models with LLM-guided evidence, we transformed large, divergent in silico predictions into a small set of four actionable candidates. We obtained experimental evidence for all four AI-prioritized candidates–one via retrospective benchmarking (Dasatinib) using the eAIC model and three via prospective *in vivo* screening (Cysteine, Tranilast, and Tretinoin) using the aAIC model, demonstrating that Cysteine, Tranilast, and Tretinoin not only improved cardiac contractility (Tukey's *post hoc*: *p* < 0.01) but also normalize key molecular markers of cardiac remodeling (Dunn's *post hoc* on *nppb* expression*: p* < 0.01) *in vivo*.

## Results

### *In Silico* Prioritization and LLM-Driven Refinement

The initial screening using the three graph learning models generated a consolidated list of 150 highly ranked AIC candidates (top 50 each model). Outputs were divergent, necessitating use of LLM agent for harmonization.

Of 150 initial candidates, 66 lacked cardiomyopathy-related literature (TxGNN: 29; CompGCN: 6; RLR: 31), while 83 possessed relevant literature (TxGNN: 21; CompGCN: 44; RLR: 19). The LLM-driven pipeline evaluated these 83 drugs, calculated Rvidence Rates (*R*_*p*_, *R*_*u*_, *R*_*n*_), and classified them based on predefined decision thresholds: Criterion 1 (Conservative/High Confidence) identified 17 potential therapeutics, whereas Criterion 2 (Sensitive/Novelty-Oriented) identified 27 (Table 1).

To isolate novel candidates for prospective validation, we applied a sequential filtering to the 27 Criterion 2 therapeutics. First, we cross-referenced the LLM classifications against the ClinicalTrials.gov and the retrieved literature. This analysis revealed that 17 of the 27 candidates had already been tested in human related to AIC (eg., Propranolol, Bosentan, Acetylcysteine), and a further 6 possessed registered cardiomyopathy-related trials. In total, 23 had existing clinical or preclinical evidence, providing external validation that the framework preferentially identifies drugs with established cardiac relevance. After excluding these, four candidates remained with no clinical trials and no human studies: Cysteine, Dasatinib, Tranilast, and Tretinoin (bold in Table 1). Notably, Dasatinib had been independently validated as cardioprotective in a previous eAIC zebrafish screen, serving as a built-in retrospective benchmark. The remaining three (Cysteine, Tranilast, and Tretinoin) represented novel, untested compounds and were advanced to prospective *in vivo* testing in the aAIC model.

As shown in Table 1, the four prioritized candidates (Cysteine, Dasatinib, Tranilast, and Tretinoin) exhibited distinct evidence profiles, illustrating the complementary utility of the dual-criterion design. Three candidates (Cysteine, Dasatinib, and Tranilast) were “Potentially Therapeutic” under both criteria. Cysteine (CompGCN, rank 3) had the largest literature corpus among the four (|*T*_*i*_| = 200) yet remained overwhelmingly neutral (*R*_*u*_(*i*) = 0.98), with a small positive signal (*R*_*p*_ = 0.02 and *R*_*n*_ = 0). Dasatinib (CompGCN, rank 21) and Tranilast (TxGNN, rank 23) each had smaller literature corpora (|*T*_*i*_| = 11 and |*T*_*i*_| = 16, respectively) and high neutrality (*R*_*u*_(*i*) = 0.91 and *R*_*u*_(*i*) = 0.94). Still, their positive evidence rates (*R*_*p*_ = 0.09 and *R*_*p*_ = 0.06) exceeded their negative rates (*R*_*n*_ = 0), securing their classification under both criteria. In contrast, Tretinoin (CompGCN, rank 41) showed a discordant profile, highlighting the value of the sensitive Criterion 2. Under the conservative Criterion 1, Tretinoin was flagged as “Potentially Adverse Rffect” because its negative evidence rate (*R*_*n*_ = 0.07) exceeded the positive rate (*R*_*p*_ = 0.03), reflecting literature on retinoid-associated cardiac toxicity (Supplementary Table 2). Due to the condition of *R*_*p*_ > 0, Criterion 2 recognised the presence of therapeutic evidence and retained Tretinoin as a candidate. This rescue mechanism proved critical subsequently. Tretinoin was then validated as cardioprotective in the aAIC model (see below), demonstrating that the dual-criterion approach prevents premature exclusion of mechanistically complex drugs with mixed evidence profiles.

### Validation of the LLM-Driven Framework Using a Zebrafish eAIC Model

To validate the translational reliability of our computational pipeline prior to prospective testing, we benchmarked outputs against a published zebrafish eAIC dataset. Wang et al. screened 37 FAAs in an eAIC zebrafish model and identified 18 FAAs with significant therapeutic effects^16^, ranked by a composite score of survival rate, fractional shortening, heart rate, and phenotypic rescue. Cross-referencing our 150-drug pool against these 18 therapeutics captured five: Spironolactone, Pravastatin, Digoxin, Dasatinib, and Valproic acid. Of these, three (Spironolactone, Digoxin, and Dasatinib) were further classified as “Potentially Therapeutic” under Criterion 2, while Pravastatin and Valproic acid were present in the candidate pool but not prioritized as therapeutic (Supplementary Table 1). These three convergent drugs (Spironolactone, Digoxin, and Dasatinib) ranked 1st, 6th, and 12th, respectively, among the 18 effective FAAs in the original eAIC screen, confirming that our framework preferentially recovers topperforming cardioprotective agents. This concordance between independent computational and experimental approaches supports the AI pipeline’s utility as a scalable screening tool.

### Translation from LLM-Driven Framework to *In Vivo* Zebrafish aAIC Model

Building on retrospective validity, we conducted prospective testing of novel *in silico* candidates. Our computational pipeline ultimately prioritized four drugs for AIC: Cysteine, Dasatinib, Tranilast, and Tretinoin. Because Dasatinib was already supported by eAIC experimental evidence^16^, we utilized the aAIC model to evaluate Cysteine, Tranilast, and Tretinoin.

Doses were determined via BSA-based interspecies conversion^17,18^. Drugs were administered by oral gavage every other day from 14 to 28 dpi, with the final DMSO concentration kept below 1%^19^. Following the 14-day treatment regimen, all three candidate drugs demonstrated robust cardioprotective effects (Figure 1A). High-frequency echocardiography revealed that Cysteine, Tranilast, and Tretinoin each significantly rescued the declined RF in DOX-injected fish (one-way ANOVA: *F*(4,95) = 47.66, *p* < 0.0001 for overall comparison; Tukey's post hoc: *p* < 0.01 for all three treatments vs. DOX control; Figure 1B). Furthermore, qRT-PCR analysis confirmed that these treatments successfully normalized the aberrantly elevated transcription of *nppb* (Kruskal-Wallis: *χ*^2^(4) = 17.21, *p* = 0.0018; Dunn’s post hoc: *p* < 0.01 for all three comparisons vs. DOX control; Figure 1C), a marker for cardiac remodeling. Beyond establishing Cysteine, Tranilast, and Tretinoin as promising novel cardioprotective therapeutics for AIC, these *in vivo* results validate the predictive power and translational prospect of our LLM-driven pipeline.

## Discussion

This study demonstrates the translational utility of an AI-driven drug repurposing pipeline for AIC. Our “Union-then-Filter” approach establishes a generic, human-in-the-process translational workflow that integrates AI-based candidate generation, automated literature synthesis, and experimental testing. The pipeline addresses a central challenge in computational drug repurposing: converting vast, divergent lists of candidate drugs into a manageable group of therapeutically plausible candidates that can be advanced to real-world biological validation.

A major bottleneck in network-based drug repurposing is the “prediction explosion” generated by different graph models^7^. By combining outputs from multiple SOTA models and then applying LLM-based evidence synthesis across more than 4,000 PubMed abstracts, our framework translates raw computational predictions into an interpretable and actionable evidence base. This step is especially important for translational research, because experimental resources are limited and not all computationally prioritized drugs are suitable for *in vivo* follow-up. Our approach reduces redundancy, filters out candidates with substantial prior clinical or *in vivo* investigation, and directs attention toward agents that are both mechanistically plausible and relatively underexplored. Retrospective recovery of known high-performers (Spironolactone, Digoxin, Dasatinib) supports the validity of the prioritization logic and increases confidence of the system to identify meaningful candidates for downstream testing.

Crucially, this pipeline is designed to augment rather than replace clinical judgement. The identification of Tretinoin illustrates the necessity of human-AI collaboration and the dual-criterion strategy. Under the conservative Criterion 1, Tretinoin was flagged for potential adverse effects. However, the sensitive Criterion 2 retained it, allowing researchers to interpret its mixed literature profiles and explore underlying therapeutic signals. Its success in the aAIC model demonstrates that this nuanced approach prevents premature exclusion of mechanistically complex candidates with mixed literature profiles.

The transition from AI-based prioritization to in *vivo* testing was enabled by the zebrafish aAIC model, which provides an efficient, biologically relevant platform for translational evaluation. This model is particularly valuable because it supports rapid phenotypic screening while incorporating cardiac readouts that are also clinically meaningful in human AIC, such as ventricular RF measured by high-frequency echocardiography^20^. The zebrafish aAIC model exhibits a biphasic pathogenesis that consists of an early stage, equivalent to patients during their chemotherapy, and a later stage, equivalent to human patients who manifest reduced RF months/years postchemotherapy. Because drugs were administered during the late phase (14 to 28 dpi), all three drugs could benefit AIC patients after they manifest cardiac dysfunction, similar to autophagy-activating FAAs previously identified by us^20^. Future work should elucidate the cardioprotective mechanisms. For example, Cysteine may enhance glutathione-dependent antioxidant defenses and reduce oxidative damage^21^; Tranilast may limit fibrotic remodeling through inhibition of TGF-β–associated profibrotic signalling^22^; Tretinoin may regulate retinoid-dependent transcriptional programs involved in cardiac adaptation and metabolic homeostasis^23^. It remains to be determined whether these three drugs also exert their cardioprotective effects via activating autophagy, and whether these drugs also exert time-dependent effects on AIC, as was shown for top FAAs^20^. Future studies are also warranted to compare therapeutic efficacy among all candidate aAIC drugs for the later stage of AIC, aiming to identify the most effective drug(s) for future translational efforts.

While this framework offers a scalable pipeline that would accelerate drug discovery, it has limitations that can benefit from refinement. First, the pipeline is partially automated; future iterations can employ agentic workflow to integrate real-time clinical trial evidence alongside literature synthesis^8,24^. The current framework relies on a single knowledge graph, which may limit the breadth; integrating multiple complementary knowledge graphs and multimodal data (transcriptomic, proteomic, chemical) could strengthen robustness and generalizability. Notably, clinically important cardioprotective agents such as sodium–glucose co-transporter-2 (SGLT-2) inhibitors (e.g., empagliflozin and dapagliflozin) did not emerge among the top-ranked candidates, despite growing literature supporting their benefit against anthracycline-induced cardiotoxicity^25–27^; this likely reflects both the prediction task being restricted to the “cardiomyopathy due to anthracyclines” indication and the underlying knowledge graph not directly encoding SGLT-2 inhibitor–cardiomyopathy relationships, further motivating the integration of multiple, more comprehensive knowledge graphs. Second, although LLM's evidence summaries can guide decision-making for researchers, fully elucidating genetic mechanisms requires dedicated mechanistic studies, which can be interrogated using the efficient zebrafish aAIC model. Third, the current prioritization framework does not constitute a comprehensive assessment of candidate safety. For example, dasatinib was classified as Potentially Therapeutic and recovered as a benchmark-positive drug in the eAIC analysis, but this tyrosine-kinase inhibitor is associated with a well-documented risk of pulmonary arterial hypertension. Thus, candidates prioritized through computational analysis and experimental screening must undergo systematic evaluation of known toxicities before advancement toward clinical translation. Fourth, while zebrafish offer scalable *in vivo* screening, species differences in electrophysiology and systemic physiology mean further mammalian models validation is necessary before clinical translation^28^. Studies in rodents and, ideally, large mammals such as porcine models should be pursued, followed by clinical evaluation.

In conclusion, this study provides a collaborative translational blueprint for AI-driven prediction and human expertise to converge and accelerate the discovery of cardioprotective therapeutics. By bridging computational candidate identification with efficient *in vivo* phenotypic rescue, we identified three clinically accessible drugs with demonstrable efficacy against anthracycline-induced cardiac injury. As cancer-survivor population continues to grow and cardiotoxicity emerges as a leading cause of non-cancer morbidity, scalable and translatable approaches to cardioprotection are urgently needed. This framework offers a highly adaptable platform not only for AIC but for any disease in which repurposing of existing therapeutics can shorten the path from computational prediction to patient benefit.

## Methods

### Data Sources and Preparation

An overview of the integrated pipeline, from knowledge-graph–based candidate generation to in vivo zebrafish validation, is shown in Figure 2. We utilized PrimeKG^29^, a comprehensive biomedical knowledge graph integrating 20 resources (e.g., DrugBank, DisGeNRT). It is particularly adept at reasoning about repurposable drugs due to its extensive drug-drug relation coverage (33.0%). Textual data, including pharmacological descriptions and mechanisms of action, were extracted from DrugBank to enrich node features. For evidence synthesis, we queried PubMed API and retrieved abstracts using each drug name combined with “anthracycline-induced cardiotoxicity” and synonyms derived from DrugBank and MeSH.

### Candidate Generation Models

We employed three SOTA graph learning models to generate an initial pool of repurposing candidates: TxGNN (Zero-shot Graph Neural Network), CompGCN (Composition-based Multi-Relational Graph Convolutional Networks), and RLR (Regularized Logistic Regression based on degree-weighted path counts). Consistent with our previous framework, we aligned the evaluation logic under a Closed World Assumption. We retrained CompGCN and RLR on PrimeKG for the “indication” edge-prediction task and used the “off-the-shelf” TxGNN results, since it was already trained on PrimeKG. For each model, we extracted the top 50 ranked drug candidates based on their prediction scores for “indication” on “cardiomyopathy due to anthracyclines”. The union formed the initial candidate pool for LLM analysis.

### LLM-Driven Prioritisation and Evidence Synthesis

To resolve the divergence between model predictions and prioritize candidates with translational potential, we employed an LLM (GPT-4.1) agent^30^. For each candidate, we retrieved up to 200 PubMed abstracts published from the year 2000 onward. Using a “context-only” prompting (Supplementary File 1), the LLM classified the relationship between the drug and AIC in each abstract into one of three categories: “Positive” (therapeutic or protective), “Neutral” (not relevant or inconclusive), or “Negative” (toxicity or no efficacy). We aggregated these judgements to calculate Rvidence Rates (*R*_*p*_ for positive, *R*_*u*_ for neutral, *R*_*n*_ for negative). Candidates were stratified using two criteria established in our previous work for each drug *i*:

$${\text{Criterion}}_{1}(i)=\left\{\begin{array}{cc} \text{Neutral}\;\text{Relation} & if\;R_{u}(i)\geq 0.99,\\ \text{Potentially}\;\text{Therapeutic} & if\;R_{u}(i)<0.99\wedge R_{p}(i)\geq R_{n}(i),\\ \text{Potentially}\;\text{Adverse}\;\text{Effect} & if\;R_{u}(i)<0.99\wedge R_{n}(i)>R_{p}(i). \end{array}\right.$$

$${\text{Criterion}}_{2}(i)=\left\{\begin{array}{cc} \text{Potentially}\;\text{Therapeutic} & if\;R_{p}(i)>0,\\ \text{No}\;\text{Positive}\;\text{Sign} & \text{otherwise}. \end{array}\right.$$

Any candidate previously tested on human subjects within the retrieved corpus was flagged in LLM synthesis. We further cross-referenced all Criterion 2 candidates against the ClinicalTrials.gov API to identify drugs with existing cardiomyopathy-related trials. These steps refined the list to novel candidates and precluded redundant experiments.

### Retrospective Framework Benchmarking

Before advancing *in silico* candidates to prospective *in vivo* testing, we benchmarked our framework against an established eAIC dataset comprising 18 FDA-approved autophagy activators (FAAs) that were previously screened in the eAIC model^16^. To prevent data leakage, we excluded the source publication from the LLM’s retrieval corpus. By cross-referencing our prioritized candidates against these 18 validated FAAs, we evaluated the LLM's automated classifications against empirical outcomes. This retrospective analysis validated the computational pipeline and filtered out redundant experiments.

### Experimental Design for aAIC Model

To prospectively validate our novel candidates, we employed an established aAIC model^16^. Adult zebrafish received a single intraperitoneal DOX injection (20 mg/kg) to induce cardiotoxicity. Based on the biphasic pathology of the aAIC model, where cardiac function declines significantly by 4 weeks post-injection (wpi), candidate drugs were administered during the late phase.

### Dose Calculation for Candidate Drugs

Since the computational prioritization pipeline identifies candidate drugs from knowledge-graph and literature relationships, the dosing information was not generated. The experimental doses for in vivo testing were derived separately using interspecies scaling as follows. Drug doses used body surface area (BSA)–based interspecies dose conversion between humans and zebrafish, which is commonly used for dose translation across species^16^:

(1) $$\text{Animal}\;\text{dose}\;(mg/kg)=\text{Human}\;\text{dose}\;(mg/kg)\times \frac{K_{m}(\text{human})}{K_{m}(\text{fish})}$$

where the *K*_*m*_ values are 37 for humans and 1 for zebrafish. Drug treatment was initiated at 14 days post-doxorubicin injection (dpi) and continued until 28 dpi, by oral gavage every other day. To minimise solvent toxicity during gavage, the final dimethyl sulphoxide (DMSO) concentration was kept below 1%, which was well tolerated in zebrafish^19^.

The final doses were 50 mg/kg Cysteine (Sigma-Aldrich; dissolved in phosphate-buffered saline (PBS)),0.3 mg/kg Tranilast (Selleckchem; dissolved in 0.5% sodium carboxymethyl cellulose, CMC-Na), and 12.3 mg/kg Tretinoin (Selleckchem; dissolved in 0.5% CMC-Na). Control and model groups received 0.5% CMC-Na as the vehicle control. At 28 dpi, therapeutic efficacy was assessed by high-frequency echocardiography, with ventricular RF as the primary endpoint. As a molecular endpoint, quantitative reverse-transcription PCR (qRT-PCR) was performed on cardiac tissue to measure *nppb* transcript levels, a recognized biomarker of adverse cardiac remodeling.

### Oral Gavage in Adult Zebrafish

Oral gavage procedure was performed following a previously established protocol^16^. Fish were anaesthetized in 0.03% buffered tricaine (MS-222, Sigma-Aldrich) for approximately 3 min. After anesthesia, the mouth of the fish was gently opened and a soft catheter tube^31^ was carefully advanced ~1.0~1.5 cm into the stomach. In most cases, the catheter could be advanced smoothly; if resistance occurred, the insertion angle was slightly adjusted to facilitate entry. The drug solution was then delivered into the stomach by slowly pressing the syringe. Administered volume was kept below 1 μL per 100 mg body weight.

### Statistical Analysis

Data normality was assessed using the Shapiro-Wilk test and Quantile-Quantile plot. Comparisons among treatment groups (DOX−, DOX+, DOX+Cysteine, DOX+Tranilast, DOX+Tretinoin) were performed using one-way analysis of variance (ANOVA), followed by Tukey's *post hoc* test or Kruskal-Wallis followed by Dunn’s *post hoc* based on the normality test. A two-sided p < 0·05 was considered significant. Data analyses were carried using GraphPad Prism^32^.

## Figures and Tables

**Figure 1:**
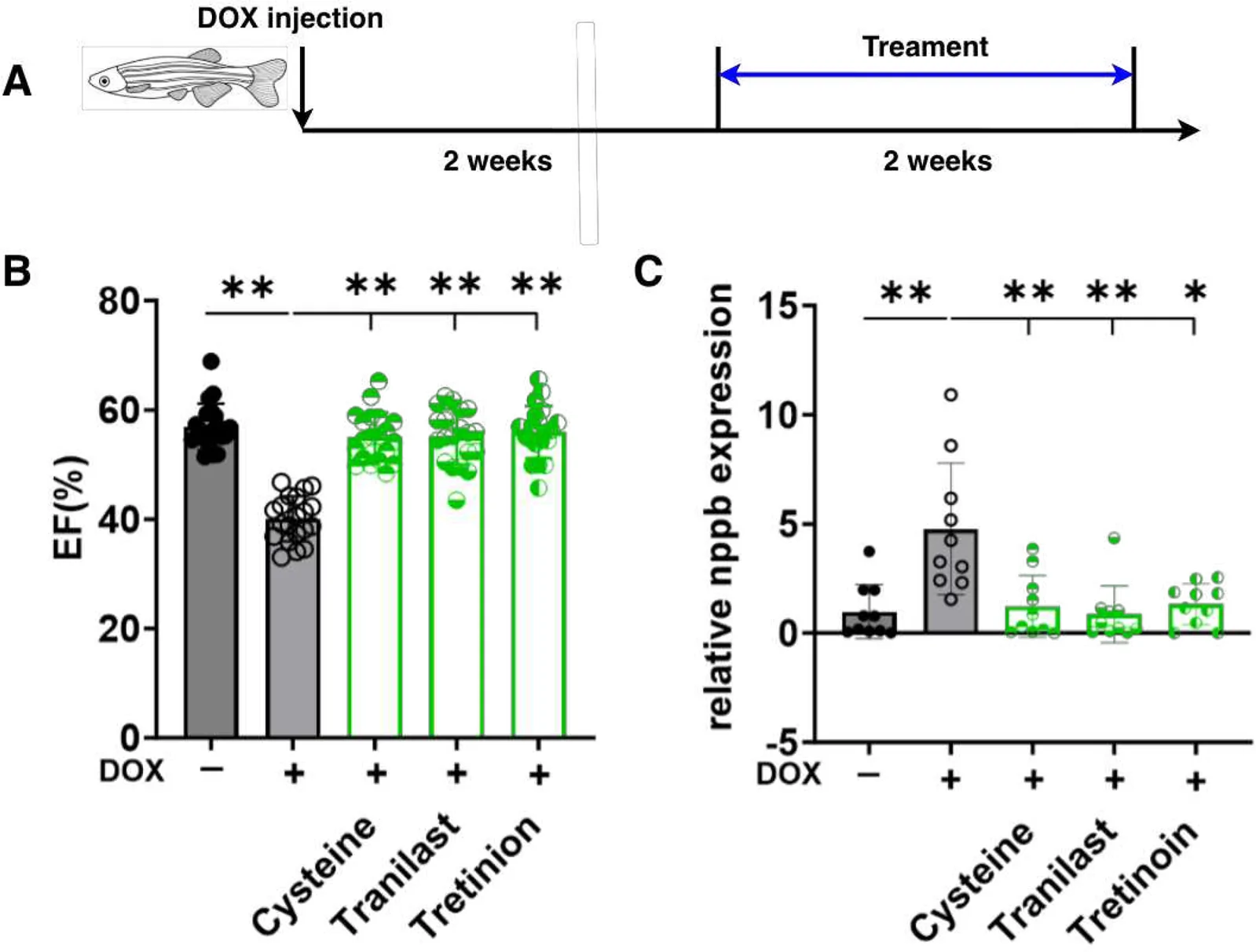
*In vivo* validation and phenotypic rescue by candidate drugs prioritized by AI for AIC. (A) Schematic representation of the experimental timeline in the adult zebrafish (aAIC) model, showing doxorubicin (DOX) injection followed by a 14-day treatment period. (B) Utilizing the adult zebrafish (aAIC) platform, the therapeutic efficacy of the AI-prioritized candidate drugs (Cysteine, Tranilast, and Tretinoin) was evaluated by ventricular ejection fraction (RF) measured using high-frequency echocardiography (n = 20). (C) Relative *nppb* mRNA expression levels were quantified by qRT-PCR to assess the molecular response to drug treatment (n = 10). Data are presented as mean ± SRM. * *P* < 0.05; **P < 0.01.

**Figure 2:**
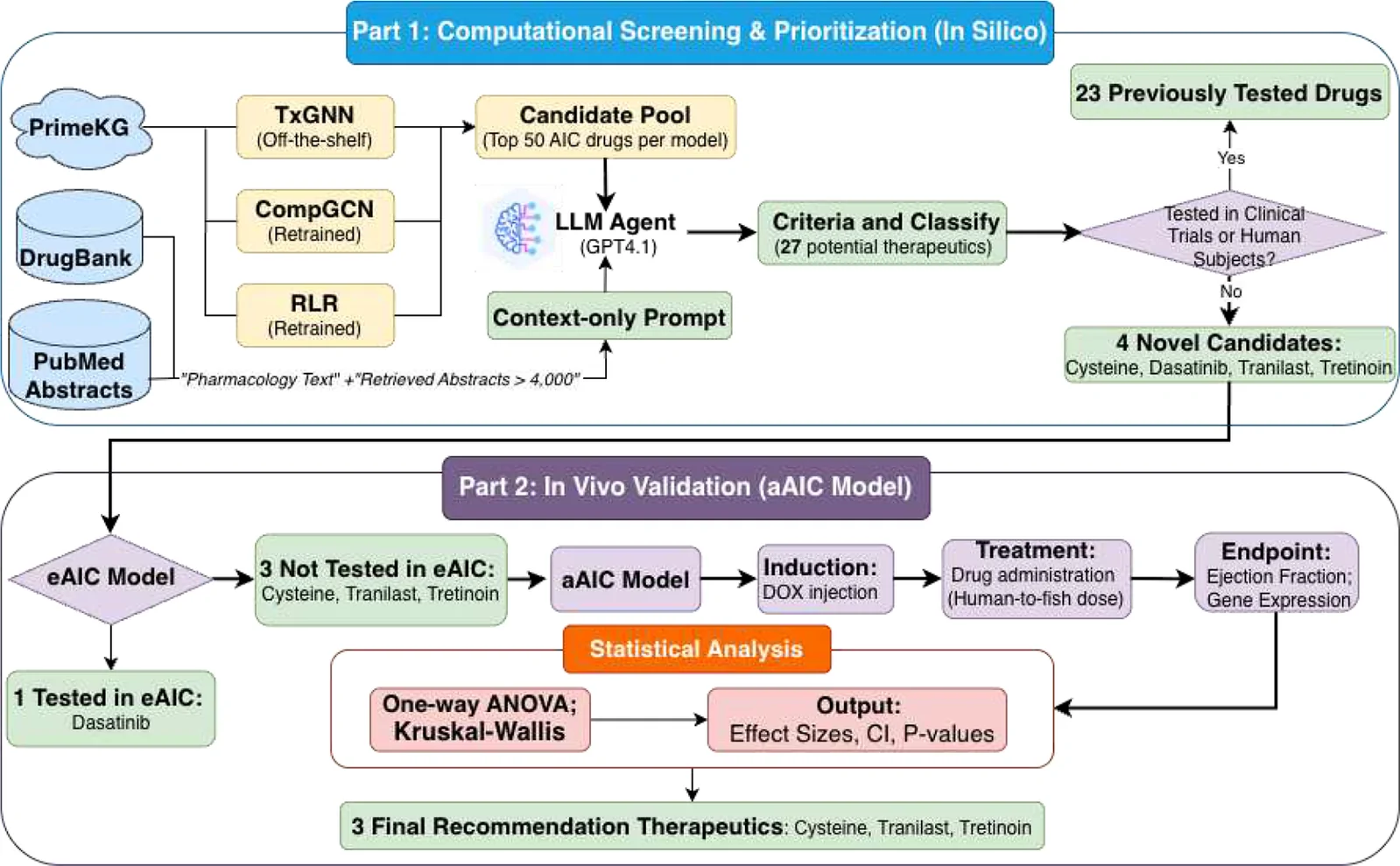
An integrated computational and in vivo framework for Anthracycline-Induced Cardiotoxicity (AIC) drug repurposing. Note: The flowchart outlines the two-part pipeline from *in silico* prediction to in *vivo* validation. Light blue nodes represent initial data sources and knowledge graphs. Yellow components denote the graph learning models used for initial candidate generation. Green indicates the LLM-driven prioritization strategy and the resulting stratified drug lists. Purple nodes represent the filtering logic and biological validation steps utilizing zebrafish models. Light red highlights the statistical testing and output metrics.

**Table 1:** Potentially therapeutic candidates prioritized by LLM-driven evidence synthesis with crossvalidation

Drug	Graph Model		LLM-driven Prioritization						Clinical Trial Count (ID)/Human Studies	Matched Trial Characterization†	
	Source	Rank	|*T*_*i*_|	*R*_*p*_(*i*)	*R*_*u*_(*i*)	*R*_*n*_(*i*)	Criterion 1	Criterion 2		Trial Type (Prevention/Treatment)	Trial Outcome
Propranolol	RLR	1	200	0.01	0.98	0.01	Potentially Therapeutic	Potentially Therapeutic	NCT05427474/Yes	Prevention (Traumatic Brain Injury)	Not reported
Bosentan	RLR	2	198	0.01	0.93	0.06	Potentially Adverse Effect	Potentially Therapeutic	0/Yes	—	—
Acetylcysteine	CompGCN	2	128	0.09	0.88	0.03	Potentially Therapeutic	Potentially Therapeutic	1(NCT01537926)/Yes	Treatment (hypertrophic cardiomyopathy)	Neutral
**Cysteine**	CompGCN	3	200	0.02	0.98	0	Potentially Therapeutic	Potentially Therapeutic	**0/No**	—	—
Vincristine	CompGCN	4	34	0.03	0.97	0	Potentially Therapeutic	Potentially Therapeutic	1(NCT06220032)/No	Prevention (anthracycline-induced cardiac dysfunction)	Ongoing
Levosimendan	RLR	7	200	0.01	0.96	0.03	Potentially Adverse Effect	Potentially Therapeutic	2(NCT02640846,NCT04166331)/Yes	Treatment (Left and Right Ventricular Function in Septic Shock)	Not reported
Ubidecarenone	RLR	10	4	0.25	0.75	0	Potentially Therapeutic	Potentially Therapeutic	3(NCT02847585,NCT01793168,etc.)/Yes	Treatment (pediatric dilated cardiomyopathy/rare diseases)	Not reported/ongoing
Glycine	CompGCN	11	129	0.01	0.98	0.01	Potentially Therapeutic	Potentially Therapeutic	1(NCT05687474)/No	Screening (genetic)	Not reported
Enalaprilat	RLR	12	57	0.02	0.96	0.02	Potentially Therapeutic	Potentially Therapeutic	9(NCT03071653,NCT04023227,etc.)/Yes	Treatment (Left Cardiac Sympathetic Denervation/chronic Chagas' cardiomyopathy)	Not reported/neutral
Enalapril	RLR	13	10	0.40	0.50	0.10	Potentially Therapeutic	Potentially Therapeutic	9(NCT03071653,NCT04023227,etc.)/Yes	Treatment (Left Cardiac Sympathetic Denervation/chronic Chagas' cardiomyopathy)	Not reported/Neutral
Erythropoietin	CompGCN	14	68	0.10	0.87	0.03	Potentially Therapeutic	Potentially Therapeutic	5(NCT00883415,NCT01493973,etc.)/Yes	Treatment (Chronic Kidney Disease/Friedreich's ataxia)	Neutral
Prednisone	RLR	15	200	0.01	0.97	0.02	Potentially Adverse Effect	Potentially Therapeutic	5(NCT01210677,NCT06635863,etc.)/Yes	Treatment (cardiac sarcoidosis/ventricular arrhythmias)	Withdrawn/ongoing
Methylprednisolone	RLR	16	200	0.01	0.96	0.03	Potentially Adverse Effect	Potentially Therapeutic	7(NCT04467411,NCT05237323,etc.)/Yes	Prevention (AIC/Myocarditis)	Ongoing/not reported
Cyclosporine	CompGCN	17	167	0.02	0.96	0.02	Potentially Therapeutic	Potentially Therapeutic	2(NCT05946772,NCT00001965)/Yes	Treatment (Takotsubo/Hypertrophic Cardiomyopathy)	Ongoing/not reported
Hydrocortisone	RLR	19	117	0.01	0.95	0.04	Potentially Adverse Effect	Potentially Therapeutic	4(NCT04425785,NCT02827799,etc.)/Yes	Treatment (Takotsubo/Insomnia in Stable Heart Failure)	Not reported
Phenytoin	CompGCN	19	7	0.14	0.86	0	Potentially Therapeutic	Potentially Therapeutic	1(NCT06465810)/No	Treatment (transthyretin amyloidosis)	Ongoing
Prednisolone	RLR	21	200	0.005	0.955	0.04	Potentially Adverse Effect	Potentially Therapeutic	7(NCT04467411,NCT05237323,etc.)/Yes	Prevention (AIC/Myocarditis)	Ongoing/not reported
***Dasatinib***	CompGCN	21	11	0.09	0.91	0	Potentially Therapeutic	Potentially Therapeutic	**0/No**	—	—
Vardenafil	CompGCN	23	7	0.14	0.86	0	Potentially Therapeutic	Potentially Therapeutic	0/Yes	—	—
**Tranilast**	TxGNN	23	16	0.06	0.94	0	Potentially Therapeutic	Potentially Therapeutic	**0/No**	—	—
Cilostazol	TxGNN	27	6	0.17	0.83	0	Potentially Therapeutic	Potentially Therapeutic	2(NCT05126836,NCT01354808)/No	Treatment (heart failure with preserved ejection fraction/acute coronary syndrome)	Positive
Ascorbic acid	CompGCN	27	64	0.09	0.88	0.03	Potentially Therapeutic	Potentially Therapeutic	3(NCT06597682,NCT02966665,etc.)/Yes	Treatment (Post-Acute Sequelae of SARS-CoV-2 Infection/Vascular Function)	Ongoing
*Digoxin*	CompGCN	36	200	0.01	0.94	0.05	Potentially Adverse Effect	Potentially Therapeutic	4(NCT04893629,NCT07238452,etc.)/No	Treatment (Cardiomyopathy / Heart Failure)	Withdrawn / ongoing
*Spironolactone*	CompGCN	37	164	0.04	0.95	0.01	Potentially Therapeutic	Potentially Therapeutic	13(NCT03071653,NCT03344159,etc.)/No	Treatment (Cardiomyopathy/right Heart Failure)	Withdrawn / not reported
**Tretinoin**	CompGCN	41	58	0.03	0.90	0.07	Potentially Adverse Effect	Potentially Therapeutic	**0/No**	—	—
Dexamethasone	CompGCN/RLR	49/20	200	0.04	0.88	0.08	Potentially Adverse Effect	Potentially Therapeutic	1(NCT03044353)/Yes	Treatment (cardiac amyloid)	Withdrawn
N-Acetylglucosamine	RLR	50	71	0.01	0.96	0.03	Potentially Adverse Effect	Potentially Therapeutic	0/Yes	—	—

Note: Bold entries denote drugs with no prior clinical trials or human studies, representing novel candidates for prospective testing. Italic entries denote drugs previously tested in the eAIC model. We surveyed publicly registered trials on ClinicalTrials.gov (e.g., NCT05427474) related to the drugs predicted by our framework to provide contextual evidence from prior literature. These trials were not part of our dataset or analyses, and no participants from these trials were enrolled or contacted.

† The Trial Type and Trial Outcome columns describe the single representative ClinicalTrials.gov record shown in the adjacent column. These records were retrieved by matching drug name to cardiomyopathy-related trials and, in most cases, concern conditions other than AIC (e.g., Takotsubo syndrome, cardiac amyloidosis, dilated cardiomyopathy) or list the drug only as a co-administered agent; “Ongoing”, “Withdrawn”, and “Not reported” indicate that no efficacy outcome has been posted for the representative trial. An em dash (—) denotes drugs with no registered trial available for classification.

## Data Availability

Data from PrimeKG are available at https://dataverse.harvard.edu/dataset.xhtml?persistentId=doi:10.7910/DVN/IXA7BM. The datasets generated and analyzed during the current study, including graph model outputs, LLM evidence classifications, are available at https://github.com/maggielee1111/LLM-Validation-AIC. An interactive platform for exploring the prioritized candidates and LLM-driven evidence summaries is publicly available at prior-rx.tech. Individual-level zebrafish experimental data (echocardiography measurements and qRT-PCR values) will be made available upon reasonable request to the corresponding authors. No individual participant data (as defined for human studies) were collected. Data will be available from the date of publication with no end date. Access to the publicly available resources requires no registration; access to additional data requires a reasonable request to the corresponding authors, who will respond within 30 days.

## Code availability

The underlying code for this study is available via this link https://github.com/maggielee1111/LLM-Validation-AIC.
